# Iron Accumulation in Deep Cortical Layers Accounts for MRI Signal Abnormalities in ALS: Correlating 7 Tesla MRI and Pathology

**DOI:** 10.1371/journal.pone.0035241

**Published:** 2012-04-17

**Authors:** Justin Y. Kwan, Suh Young Jeong, Peter Van Gelderen, Han-Xiang Deng, Martha M. Quezado, Laura E. Danielian, John A. Butman, Lingye Chen, Elham Bayat, James Russell, Teepu Siddique, Jeff H. Duyn, Tracey A. Rouault, Mary Kay Floeter

**Affiliations:** 1 National Institute of Neurological Disorders and Stroke, National Institutes of Health, Bethesda, Maryland, United States of America; 2 Molecular Medicine Program, National Institute of Child Health and Human Development, National Institutes of Health, Bethesda, Maryland, United States of America; 3 Advanced MRI Section, Laboratory of Functional and Molecular Imaging, National Institute of Neurological Disorders and Stroke, National Institutes of Health, Bethesda, Maryland, United States of America; 4 Feinberg School of Medicine, Northwestern University, Chicago, Illinois, United States of America; 5 National Cancer Institute, National Institutes of Health, Bethesda, Maryland, United States of America; 6 Radiology and Imaging Sciences, Clinical Center, National Institutes of Health, Bethesda, Maryland, United States of America; 7 Department of Neurology, George Washington University, Washington, D. C., United States of America; 8 Department of Neurology, University of Maryland, Baltimore, Maryland, United States of America; University of Florida, United States of America

## Abstract

Amyotrophic lateral sclerosis (ALS) is a progressive neurodegenerative disorder characterized by cortical and spinal motor neuron dysfunction. Routine magnetic resonance imaging (MRI) studies have previously shown hypointense signal in the motor cortex on T_2_-weighted images in some ALS patients, however, the cause of this finding is unknown. To investigate the utility of this MR signal change as a marker of cortical motor neuron degeneration, signal abnormalities on 3T and 7T MR images of the brain were compared, and pathology was obtained in two ALS patients to determine the origin of the motor cortex hypointensity. Nineteen patients with clinically probable or definite ALS by El Escorial criteria and 19 healthy controls underwent 3T MRI. A 7T MRI scan was carried out on five ALS patients who had motor cortex hypointensity on the 3T FLAIR sequence and on three healthy controls. Postmortem 7T MRI of the brain was performed in one ALS patient and histological studies of the brains and spinal cords were obtained post-mortem in two patients. The motor cortex hypointensity on 3T FLAIR images was present in greater frequency in ALS patients. Increased hypointensity correlated with greater severity of upper motor neuron impairment. Analysis of 7T T_2_^*^-weighted gradient echo imaging localized the signal alteration to the deeper layers of the motor cortex in both ALS patients. Pathological studies showed increased iron accumulation in microglial cells in areas corresponding to the location of the signal changes on the 3T and 7T MRI of the motor cortex. These findings indicate that the motor cortex hypointensity on 3T MRI FLAIR images in ALS is due to increased iron accumulation by microglia.

## Introduction

A reliable objective marker of corticospinal upper motor neuron (UMN) dysfunction remains elusive despite its importance for diagnosis and monitoring of disease progression in amyotrophic lateral sclerosis (ALS). Clinical signs of UMN impairment such as pathological reflexes and increased tone on neurological examination remain the mainstay for detecting cortical neuronal loss and corticospinal tract degeneration in motor neuron disease [1]. Reduction in the neuronal marker N-acetyl-aspartate (NAA) on proton magnetic resonance spectroscopy, decreased fractional anisotropy on diffusion tensor imaging studies, and prolongation of central motor conduction time on transcranial magnetic stimulation studies have been suggested as potential markers of corticospinal motor neuron dysfunction in ALS [2]–[4]. While these measures facilitate an improved understanding of the underlying pathophysiology, they are neither sensitive nor specific for ALS. Furthermore, there is no definite correlation between these imaging and electrophysiological parameters and clinical manifestations of UMN impairment.

Conventional magnetic resonance imaging (MRI) is routinely performed during the evaluation of patients suspected of having ALS to exclude other diagnoses that may mimic this condition. Several MRI signal alterations in the precentral gyrus have been noted in ALS patients, including hyperintense signal in the subcortical white matter and hypointense signal in the grey matter on T_2_-weighted and fluid attenuated inversion recovery (FLAIR) MR images [5]. Motor cortex hypointensity correlated with the severity of clinical UMN dysfunction in one study, and was proposed as a marker of motor neuron degeneration [6]. However, hypointensity on T_2_-weighted MRI of the motor cortex is inconsistently present in ALS patients, and its specificity for neuronal degeneration in ALS is uncertain [7], [8]. T_2_ shortening on MRI of the motor cortex has been seen in a few other chronic conditions, mainly in patients over 70 years old [9]. The underlying cause of the T_2_ signal reduction (i.e. reduction of the T_2_ relaxation time constant) in the motor cortex of ALS patients is not entirely clear. Iron deposition resulting from the neurodegenerative process was proposed as the cause of signal shortening [10], but an imaging sequence sensitive for detecting signal alterations caused by the local susceptibility effects of iron failed to support this hypothesis [11].

Several neurodegenerative disorders such as neurodegeneration with brain-iron accumulation and Parkinson disease are known to have excessive iron accumulation that correspond to signal alterations on the MR images in various brain regions [12]. In these diseases, excessive iron accumulation is hypothesized to cause neuronal damage. In ALS, abnormal iron homeostasis inducing excessive oxidative stress in the motor neurons has been postulated to contribute to disease pathogenesis [13], based on several lines of evidence. For example, axotomy led to elevated expression of transferrin 1 receptor, resulting in increased iron uptake into the motor neuron [14]. Quantitative evaluation of iron demonstrated elevated iron levels particularly in the nucleus of motor neurons from the cervical spinal cord of ALS patients, leading to the speculation that increased intraneuronal iron may confer susceptibility to degeneration in ALS [15]. In addition, elevated transcripts of ferritin heavy and light subunits were observed in the spinal cord of the superoxide dismutase 1 (SOD1) G93A mouse model of familial ALS [16]. The observation that iron accumulates in SOD1 G37R mouse spinal neurons and glia, and that there is a delay in the progression of disease with iron chelator treatment in SOD1 G93A mice further suggested a pathogenic role of iron in familial ALS [17], [18]. A number of recent studies have investigated the association of polymorphisms in the HFE gene with ALS. Alteration in the coding region of both alleles of the HFE gene causes the autosomal recessive iron overload syndrome hereditary hemochromatosis [19]. Increased frequency of the H63D variant of HFE has been associated with neurodegenerative disorders, including ALS, further suggesting a role of abnormal iron metabolism in ALS [20]–[23]. The association between the HFE genotype and ALS, however, has not been observed in all populations in genome wide association studies [24], [25]. Whether iron misregulation or accumulation in motor neurons has a causative role in ALS has not been established. As an early step to investigate the role of iron in ALS, neuroimaging methods can be used to confirm and localize iron deposition in the cortex of ALS patients.

Iron deposition in tissue causes a concentration-dependent irregularity in the local magnetic field and signal loss during MRI acquisition because iron deposits become magnetized and cause the surrounding water molecules to both accumulate phase and lose phase coherence [26], [27]. This property of iron has led to the use of T_2_-star (T_2_^*^) weighted gradient-echo sequences to detect the presence of iron in different tissues with greater sensitivity [26], [28]. Hemosiderin, deoxyhemoglobin, ferritin, calcium, and air can all cause phase shifts and signal intensity loss on T_2_^*^ weighted gradient-echo images because of local alterations in the magnetic susceptibility [29]. In MRI with a higher field strength, the enhanced T_2_^*^ contrast further increases the sensitivity for detecting increased iron, as well as provides higher spatial resolution [30]. The purpose of this study was twofold: to better understand the anatomical basis of the MR signal changes in the motor cortex in ALS patients through correlation of pathology to high-field MR images, and to determine whether hypointensity in the motor cortex on the T_2_-weighted FLAIR MR images can be used as a marker for degeneration of upper motor neurons.

## Methods

### Ethics Statement

This protocol was approved by the NIH Combined Neuroscience Institutional Review Board. All subjects gave written, informed consent for the protocol.

### Subjects

Nineteen patients with probable or definite ALS by El Escorial criteria [31], and 19 age-matched healthy controls participated in the protocol. The ALS patients were prospectively recruited over a two-year period from two regional academic ALS centers. The patients also participated in diffusion tensor imaging studies that were previously reported [4]. All subjects underwent 3T MRI of the brain. A subset of five ALS patients and three controls also had 7T MRI studies.

### Clinical evaluation

A detailed history and neurological examination was obtained by a neurologist in all subjects. All patients underwent diagnostic testing to exclude other diseases that may mimic motor neuron disease and to confirm that they fulfilled the El Escorial diagnostic criteria for clinically probable or definite ALS. Disease severity was graded using the revised ALS functional rating scale (ALSFRS-R) in all subjects. The severity of upper motor neuron dysfunction was quantified using the upper motor neuron impairment score (UMN-IS) which was calculated from clinical scales for grading reflexes [32] and muscle tone [33], and from timed measurements of fingertapping, gait, and speech, allowing for side- or limb-specific measures. A composite score from 0–5 was generated for each limb and the cranial region, with normal function = 0, and higher scores indicating greater UMN impairment [4]. All healthy controls had a normal neurological examination and ALSFRS-R.

### 3T Neuroimaging Acquisition

In all subjects, MRI of the brain was performed on a 3.0 tesla (T) scanner (Philips Achieva, Best, the Netherlands) using a receive-only, 8-channel head coil. 2D FLAIR imaging data was obtained. Parameters for the FLAIR imaging sequence were: 57 axial slices, 2.5 mm slice thickness interleaved with no gap and 1 excitation, TE = 140 ms, TR = 11000 ms, TI = 2750 ms, echo train length 20, 320×219 acquisition matrix and 220 mm field of view.

### 7T Neuroimaging Acquisition (*In Vivo*)

Five ALS patients who did not have significant respiratory or bulbar impairment that precluded participation in additional MRI studies and whose 3T scans showed hypointensity on the FLAIR imaging in the hand knob region of the motor cortex, and 3 healthy controls underwent additional MRI at 7.0 T (Signa scanner, GE Healthcare) equipped with a Nova Medical transmit coil and 32-channel receive array. The following contrast parameters were used: dual gradient echo acquisition with TE = 14 and 28.5 ms, bandwidth = 62.5 kHz, TR = 1 s, flip angle 60 degrees. The geometric parameters were: 20 slices at 1 mm slice thickness with 0.5 mm gap, about 15 degrees oblique with respect to axial, 768×576 matrix with SENSE rate 2 acquisition, and nominal resolution of 312×312 mm^2^.

### 7T Neuroimaging Acquisition (*Ex Vivo*)

In one ALS patient for whom 3T and 7T MRI were obtained one year prior to death, one half of the postmortem brain was sliced in the axial plane for imaging on the 7.0 T Signa scanner using a Nova Medical volume transmit coil and with a 24-element receive-only detector array custom built for tissue scanning. Contrast parameters were: four gradient echo acquisitions with TE = 23, 30, 37, and 44 ms, bandwidth = 250 kHz, TR = 1 s, flip angle 55 degrees. Geometric parameters were: 3D acquisition, 1024×512×160 matrix, and nominal resolution of 175×175×175 mm^3^.

### Neuroimaging Analysis

Each 3T FLAIR sequence was visually assessed by two independent investigators blinded to the subject's diagnosis. In each hemisphere, the motor cortex area corresponding to the hand knob [34], was identified. The magnitude of hypointensity on the FLAIR images within the hand knob was compared to the adjacent postcentral cortex in several axial slices. Signal hypointensity was graded as 0 (absent), 1 (present, mild), or 2 (present, marked). A preliminary study evaluating the inter-observer agreement of motor cortex hypointensity showed an agreement of 76±5%.

Gradient echo magnitude images, frequency and R_2_^*^ maps on 7T were visually inspected for signal alteration in the motor cortex and other cortical and subcortical regions. For 7T images, investigators were not blinded to the subject's diagnosis. Decreased signal on the magnitude scans and increased signal on R_2_^*^ maps were graded as 0 (absent), 1 (present, mild), or 2 (present, marked). Signal changes on the magnitude scans and R_2_^*^ maps in the precentral gyrus were evaluated and compared to other cortical (cingulate gyrus) and subcortical areas (basal ganglia). In all 7T imaging studies, the R_2_^*^ maps were calculated by taking the inverse logarithm of the ratio of the first and second echo. The result was divided by the echo time difference to obtain a per-second (Hz) scale. R_2_^*^ was only calculated in areas with sufficient signal.

### Postmortem examination

The brains and spinal cords of two ALS patients, ages 42 and 51, were removed postmortem in their entirety and were fixed in formalin. In both patients, the motor cortex hypointensity had been scored as grade 2 on 3T images, and the R_2_* signal was marked on 7T images. Representative sections of the spinal cord, motor cortex, and other cortical regions from one hemisphere were processed for standard clinical pathology in six micron thick paraffin embedded sections. The other hemisphere was used for additional histochemical and immunocytochemistry studies and post-mortem imaging studies. Following post-mortem imaging, tissue blocks were obtained from the regions identified as the pre-and post-central gyrus in the hand knob region, premotor cortex, medial orbitofrontal cortex and the occipital cortex. As control for the histological stains, a block of motor cortex from an age, gender, and ethnicity-matched control with no known neurological disease was obtained from the New York Brain Bank—The Taub Institute, Columbia University. Two blocks of motor cortex from an 85-year-old patient diagnosed with idiopathic Parkinson disease and a 77-year-old patient with Alzheimer disease were obtained from the NIH Laboratory of Pathology as neurological disease controls.

Sections from the ALS patients and the control cortex were stained with hematoxylin and eosin, Bielchowsky's silver stain, and luxol fast blue/cresyl violet. Histochemical stains for aluminum, calcium, and copper were carried out on one of the ALS patient's tissue, as were immunohistochemical stains for ubiquitin, glial fibrillary acidic protein (GFAP), TDP-43 antibody, phosphorylated TDP-43, and FUS, using methods previously described [35]. Immunocytochemistry for TDP-43 was also carried out on the control subject's cortex. The sections were deparaffinized and rehydrated by passing the slides in serial solutions: 3× for 10 minutes each in xylene, 3× for 5 minutes each in 100% ethanol, 3× for 3 minutes each in 95% ethanol, once for 5 minutes each in 75% ethanol, 50% ethanol, deionized water, and phosphate-buffered saline (PBS). The antigens in the sections were retrieved using a decloaking chamber using a retrieval solution (10 mM citrate acid, pH 6.0) at 125°C for 20 minutes. The sections were cooled to room temperature for 30 minutes and rinsed with deionized water for 5 minutes. The endogenous peroxidase activities were blocked with 2% hydrogen peroxide. Non-specific background was blocked with 1% bovine serum albumin in PBS for 20 minutes at room temperature. Primary antibodies included those to FUS (11570-1-AP, 1∶50 dilution, Proteintech Group, Chicago, IL), TDP43 (10782-2-AP, 1∶4,000 dilution, Protein Tech Group, Chicago, IL), phosphorylated TDP43 (TIP-PTD-P01, 1∶5,000 dilution, Cosmo Bio Co, Tokyo, Japan) and ubiquitin (PRB-268C, 1∶4,000 dilution, Covance, Emeryville, CA). Primary antibodies were applied to the slides and incubated at room temperature for 1 hour. After rinsing the slides 3× for 5 minutes each, a biotinylated goat anti-rabbit or anti-mouse immunoglobulin G (IgG) was applied to the slides as the secondary antibody (GR608H or GM601H, Biocare Medical, Concord, CA). The slides were incubated at room temperature for 30 minutes. The excess secondary antibody was removed by rinsing the slides 3×, 5 minutes for each time. The signals were detected with peroxidase-conjugated streptavidin (BioGenex, San Ramon, CA) using 3-amino-9-ethylcarbazole as a chromogen. The slides were counterstained with hematoxylin and sealed with Aqua Poly/Mount (Polyscience, Warrington, PA). Perls' DAB staining for iron, and immunostaining for ferritin and CD68, a marker for macrophage/microglial cells, were performed on sections of the motor cortex, premotor cortex, medial orbitofrontal cortex, and lumbar spinal cord using previously described methods with some modifications [36]. Briefly, sections were treated with xylene to remove paraffin and rehydrated. After PBS washes, sections were incubated in 2% potassium ferrocyanide solution with 2% hydrochloric acid at room temperature for 1 hour. After washing, sections were incubated in unactivated DAB solution with 0.01% nickel chloride followed by the same solution with 0.03% H_2_O_2_ for 20 minutes each. For double immunofluorescent staining, deparaffinized sections were blocked with 4% normal goat serum, 2% ovalbumin and unlabeled goat anti-mouse IgG (50 ug/ml, Jackson Laboratories). Rabbit anti-ferritin (1∶1,000 dilution, kind gift of Dr. Esther Meyron-Holtz, Technion, Haifa, Israel) and mouse anti-CD68 (1∶500 dilution, Serotec) were applied to the slides and incubated overnight. On the next day, the sections were washed and the signals were visualized by incubating with Alexa Fluor 488/546 conjugated secondary antibodies for one hour. Slides were mounted with anti-fading media containing DAPI (Vectashield mounting media, Vector Lab) to visualize the nucleus. Perls' DAB staining and immunocytochemistry for CD68 and ferritin were also carried out on the motor cortex from the control, Parkinson disease, and Alzheimer disease.

### Statistical Analysis

All data are presented as mean ± standard deviation. In all tests used to analyze clinical parameters and cortical hypointensity, the statistical significance threshold was set at p<0.05 corrected, unless otherwise specified. Statistical analysis was performed using SPSS Statistics version 17.0 and SAS Enterprise Guide version 4.3.

## Results

### Demographics

Seven ALS patients fulfilled the revised El Escorial criteria for clinical definite ALS and 12 patients had clinical probable ALS. The average ALSFRS-R scores was 31 (S.D. = 6) in clinical definite ALS patients and 38 (S.D. = 6) in clinical probable ALS patients. Age and gender did not differ between ALS patients and controls. The ALSFRS-R was lower and UMN-IS was higher in the patient group compared with the controls (Table 1).

**Table 1:** Clinical Features of ALS Patients and Healthy Controls.

	ALS	HC	ALS 7T
**N**	19	19	5
**Gender (Male∶Female)**	9∶10	11∶8	4∶1
**Age, years**	53.1±9.4	58.7±5.9	49.8±8.6
**Disease duration (range), years**	2.3±1.4 (0.5–5.0)		1.8±1.2
**ALSFRS-R**	35.7±6.8*	48.0	38.0±7.0*
**UMN-IS**	1.96±0.80*	0.05±0.07	1.76±0.82*

* ALS significantly different from healthy controls (HC).

### 3T Neuroimaging Analysis

**Figure 1:**
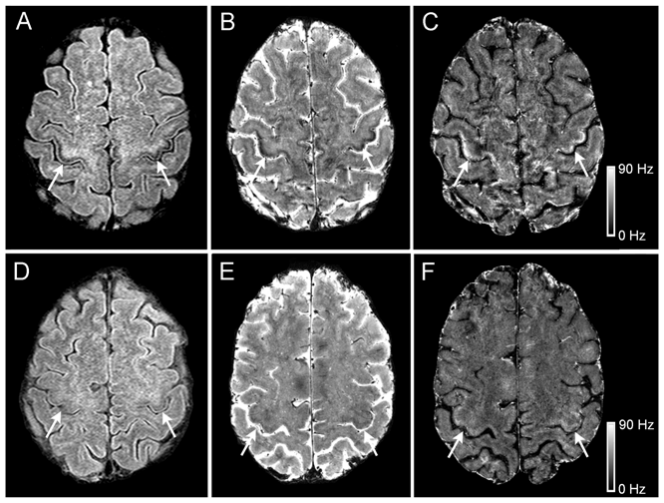
3T and 7T MRI in ALS and healthy control. 3T and 7T brain MRI signal changes in the motor cortex hand knob differed between ALS patients (A, B, C) and healthy controls (D, E, F). Cortical hypointensity (*arrows*) is shown to be present in an ALS patient (Patient 2, age 51) and not in a healthy control on 3T FLAIR (A, D) and 7T gradient echo magnitude images (B, E). These areas corresponded to hyperintensity in the 7T R_2_^*^ maps (C, F). Scale bar represents R_2_^*^ value in Hz.

**Figure 2:**
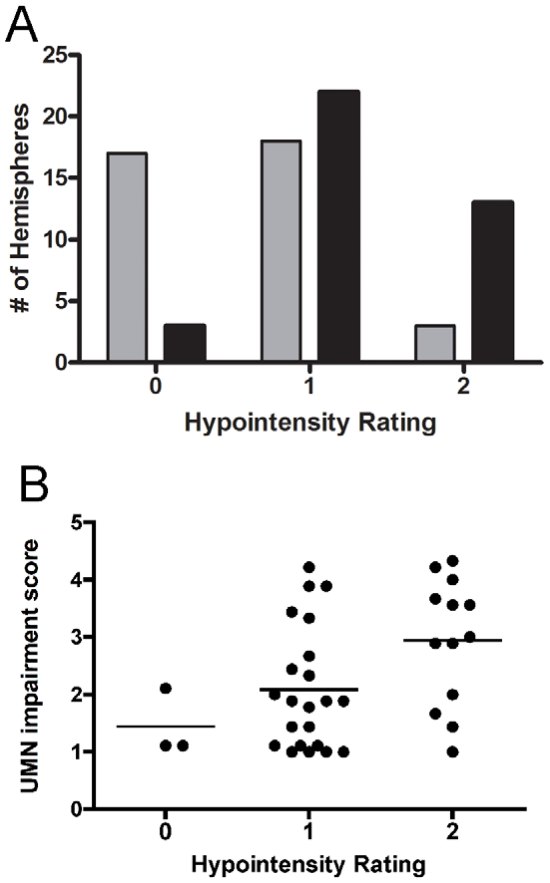
Frequency of hypointensity grade in the hand knob of the motor cortex. (A) Higher grades of hypointensity in the hand knob region on 3T FLAIR MRI occurred with greater frequency in ALS patients (*black bars*) than in healthy controls (*gray bars*). (B) Higher grades of hypointensity on 3T FLAIR MRI was associated with higher upper motor neuron impairment score (UMN-IS). Each dot represents the grade for one hemisphere and the UMN-IS score for the contralateral limb. Line indicates mean for group. Hypointensity was rated grade 0 (absent), grade 1 (present, mild), and grade 2 (present, marked). The UMN-IS is graded from 0–5 with normal function = 0, and higher scores indicating greater UMN impairment.

On the 3T axial FLAIR MRI sections in ALS patients, a curvilinear band of decreased signal was identified along the anterior banks of the central sulcus in the precentral gyrus of both hemispheres following the gyral pattern and extending into the parasagittal region. The signal was deep within the cortical grey, with a patchy appearance (Fig. 1A, arrows). Similar patterns were not consistently seen in FLAIR images of controls (Fig. 1D, arrows). Compared to healthy controls, more ALS patients had higher grades of hypointensity on FLAIR images in the hand knob of the motor cortex (Fig. 2A). In the 38 ALS hemispheres, the hand knob hypointensity was graded as marked (grade 2) in 13, mild (grade 1) in 22, and absent (grade 0) in 3. In contrast, hand knob hypointensity on FLAIR images in healthy control hemispheres were grade 0 in 17, grade 1 in 18, and grade 2 in only 3. ALS was associated with higher hypointensity grades on the FLAIR images (Fisher's exact test, p<0.0001), and the incidence of ALS increased with increasing hand knob hypointensity (Cochran Armitage trend test, p<0.0001). Increasing hand knob hypointensity grade significantly correlated with greater motor impairment as determined by the limb specific UMN-IS (r = 0.56, p<0.01; Fig. 2B) and the ALSFRS-R (r = −0.41). The hand knob hypointensity score did not correlate with disease duration.

### 7T Neuroimaging Analysis (*In Vivo*)

Five ALS patients whose 3T FLAIR images had mild or marked hypointensity in the hand knob of the motor cortex and three controls underwent scanning on a 7T MRI. Of the 10 ALS hemispheres, 8 had grade 2 hand knob hypointensity and 2 had grade 1 hand knob hypointensity. On the 7T scan, the R_2_^*^ maps showed marked signal hyperintensity in the deeper portion of the motor cortex in four ALS patients (Fig. 1C) and mild hyperintense signal in one ALS patient. The hyperintensity extended into the parasagittal area. The hyperintense signal was discontinuous, with a patchy appearance. Two of the controls had a grade 1 hypointensity score on the 3T MRI. Similarly, there was mild decreased signal in the hand knob region on the 7T gradient echo magnitude images (Fig. 1E). Two control subjects also had mildly increased signal (Fig. 1F) and one control had markedly increased signal in the motor cortex on the R_2_^*^ map. The increased signal on the R_2_* map in control subjects was located in a similar distribution as that seen in ALS patients. In the 7T gradient echo magnitude scans, a region of decreased signal extended the entire length of the cortex in the precentral gyrus including the parasagittal area in ALS patients (Fig. 1B), primarily in the deeper portions of the cortex. In some images, the hypointensity appeared to occupy the full thickness of the cortex. Decreased signal in the gradient echo scans was not seen in the cingulate cortex of ALS patients or controls, but was present in the basal ganglia of patients and controls.

### 7T Neuroimaging Analysis (*Ex Vivo*)

**Figure 3:**
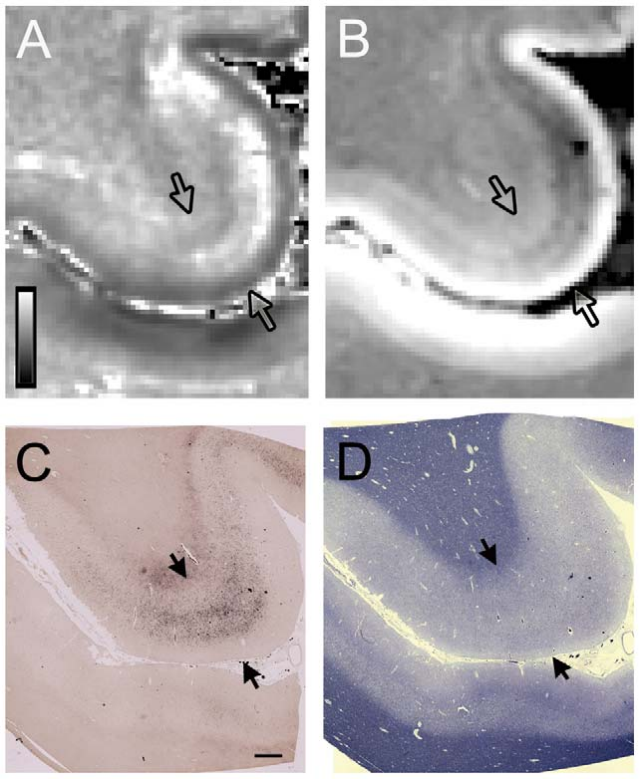
*Ex vivo* 7T MRI and corresponding pathology in ALS. 7T MRI signal change corresponded to iron accumulation in deeper layers of the cortical gray in the hand knob in an ALS patient. Each panel shows an axial slice of the precentral gyrus, with the cortical gray matter boundaries indicated by arrows, and the central sulcus and postcentral gyrus below. (A) R_2_^*^ map showed patchy hyperintensity in the precentral gray. Scale bar represents R_2_^*^ value in Hz. (B) Gradient echo magnitude image showed hypointensity in the corresponding region. Pixels correlate to acquisition voxels. (C) Low power micrographs show iron accumulation in the middle and deep layers of the motor cortex with Perls' DAB stain. (D) The luxol fast blue stain showed myelin pallor in the subcortical white matter of the precentral gyrus compared to the postcentral gyrus. Scale bar, 1 mm.

Postmortem 7T imaging of the brain was obtained in one ALS patient. The R_2_^*^ map (Fig. 3A) showed hyperintense signal in the deep layers of the grey matter of the motor cortex. The signal was patchy in some areas and was separated from white matter by a band with normal signal intensity. The gradient echo magnitude scan showed decreased signal in the same layers of the motor cortex (Fig. 3B). There was a broad band of altered signal at the gray white junction that was also present in many cortical regions beyond the motor cortex. No other cortical areas had observable signal alterations on the gradient echo magnitude scan or R_2_^*^ map within the cortical gray matter.

### Clinico-pathological Studies

**Figure 4:**
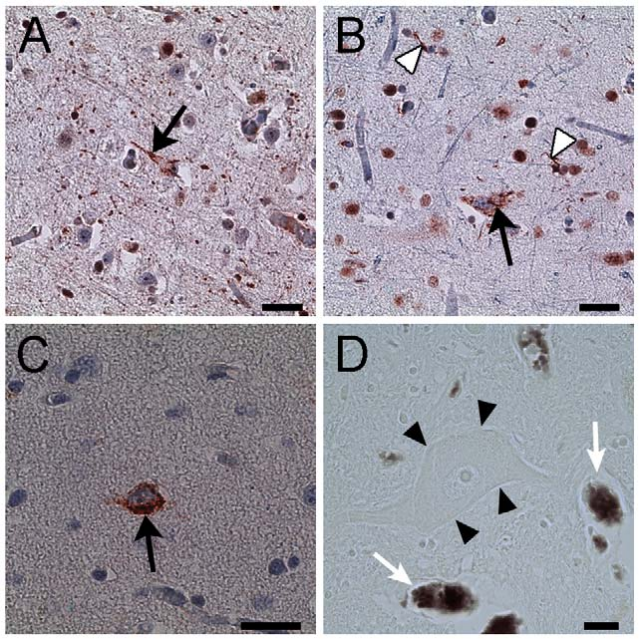
Immunohistochemistry of cellular inclusions and iron histology in an ALS patient. (A) Ubiquitin reactive neuronal cytoplasmic inclusion with a filamentous appearance (*arrow*) in motor cortical neuron. (B, C) The neuronal cytoplasmic inclusions (*arrows*) were reactive with TDP-43 (B) and phosphorylated TDP-43 (C). Glial cell inclusions (B, *white arrow heads*) were also TDP-43 positive. (D) Spinal motor neurons (*arrow heads*) did not show iron accumulation with Perls' DAB stain which was present in erythrocytes in the same section (*arrows*). Scale bar, 100 mm (A–C); 20 mm (D).

The gross pathological examination of both brains did not reveal perceptible global or frontal lobe atrophy. Histological examination of sections from the spinal cord showed typical pathology for ALS, including loss of motor neurons, reactive astrocytosis, atrophy of the anterior nerve roots, and axonal spheroids. Ubiquitin positive neuronal cytoplasmic skein-like inclusions (Fig. 4A) and Bunina bodies were present in some of the remaining neurons. In sections from the motor cortex, there were decreased numbers of large pyramidal cells in the middle and deep layers of the cortical mantle and pallor of the subcortical white matter compared to the adjacent postcentral gyrus (Fig. 3D). Immunostaining for GFAP showed reactive astrocytes. Immunostaining for TDP-43 showed focally increased cytoplasmic inclusions in some neurons and glial cells (Fig. 4B). Some neurons and glia lacked nuclear staining for TDP-43. Hyperphosphorylated TDP-43 was only found in the inclusions (Fig. 4C). No apparent FUS-immunoreactive inclusions were observed. Histochemical stains for aluminum, calcium, and copper showed no abnormal reactions.

### Iron Histology

**Figure 5:**
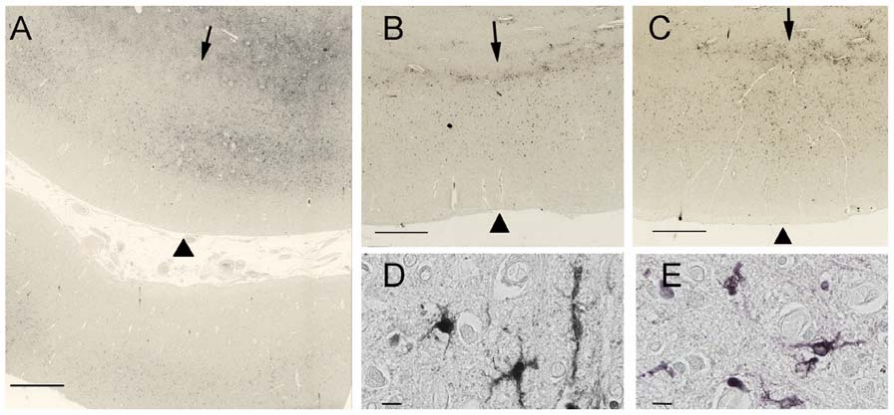
Histological staining for iron with Perl's DAB stain in the motor cortex. The panels show comparable planes of section through the precentral gyrus of brains from the second ALS patient (A), and from the Alzheimer (B) and Parkinson (C) patients. The arrowheads indicate the pial surface. The arrows indicate the gray-white junction. (A) Low power magnification showing iron accumulation in the middle and deeper layers of cortical gray matter, and at the gray-white junction in a 51-year-old ALS patient. At higher power (D, E) the staining is present in cells with irregular processes suggestive of microglia in the ALS motor cortex. The lower portion of panel A shows the post-central gyrus with relatively little iron staining. The iron staining was more diffuse within the motor cortex of Alzheimer (B) and Parkinson (C) patients and was also present in the subcortical white matter. Scale bars, 1 mm (A–C), 10 µm (D, E).

Perls' DAB staining revealed abundant cells containing intracellular iron and occasional extracellular iron deposits in the motor cortex of both ALS patients (Fig. 3C, Fig. 5A). The intracellular iron was observed in small elongated cells with irregular short processes suggestive of microglia (Fig. 5D, E). The staining was primarily located in the middle and deep layers of the motor cortex, sparing the superficial layers. In adjacent counterstained sections, these regions corresponded to a broad layer of medium-large pyramidal neurons, which was separated from a deeper cortical layer that contained a few large pyramidal neurons amid bundles of myelinated fibers (Fig. 3D).

**Figure 6:**
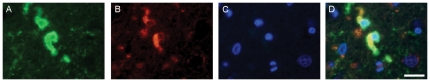
Double immunofluorescent labeling of ferritin-rich microglia in the motor cortex of an ALS patient. Immunostaining for ferritin in green (A) and CD68, a marker of microglia, in red (B) labeling showed co-localization in yellow (D, merge) indicating increased microglial ferritin in the motor cortex of an ALS patient. (C) DAPI immunofluorescence, in blue, shows nuclear labeling. Scale bar, 20 mm.

Ferritin immunostaining showed a similar distribution to the Perls' DAB staining, suggesting that most of the iron was sequestered in intracellular ferritin. Immunostaining showed increased CD68 positive cells in the deep layers of the motor cortex. Double immunofluorescence studies showed co-localization of ferritin with CD68, indicating that the ferritin was sequestered in activated microglia (Fig. 6A–D). No ferritin staining was identified in large nucleated cells in the motor cortex that were presumed to be healthy neurons. Some Perls' DAB staining was also noted in the white matter adjacent to the motor cortex. The sensory cortex had very slight Perls' DAB staining (Fig. 3C). There was no appreciable Perls' staining in the other cortical areas examined.

To evaluate the specificity of the Perls' DAB iron stains in the motor cortex, we examined tissue from a Parkinson and an Alzheimer patient. Perls' DAB staining showed some iron accumulation in the motor cortex of these patient controls, but the iron staining was more diffuse in the cortical grey than in the ALS brains, and also extended into the white matter adjacent to the cortex (Figure 5B, C). Immunofluorescence studies showed ferritin in the same distribution as the Perls' DAB staining. The ferritin immunostaining, which is more specific for intracellular iron, was less abundant than the Perls' DAB staining in the section from the Parkinson patient's brain. In the tissue from the patient with Alzheimer disease, there was slight iron staining mainly in the white matter adjacent to the cortex and a few scattered cells in the cortex (Fig. 5B). The motor cortex from the control brain had no staining with Perls' DAB staining or ferritin immunoreactivity.

**Figure 7:**
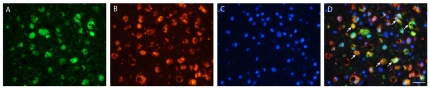
Double immunofluorescent labeling of ferritin-rich microglia in the dorsolateral white matter from the spinal cord of an ALS patient. Immunostaining for ferritin in green (A) and CD68, a marker of microglia, in red (B) labeling showed co-localization (*arrows*) in yellow (D, merge) indicating increased microglial ferritin in the corticospinal tract from the spinal cord of an ALS patient. (C) DAPI immunofluorescence, in blue, shows nuclear labeling. Scale bar, 30 mm.

In the spinal cord, many cells with increased CD68 immunoreactivity were found in the dorsolateral funiculus (Fig. 7B) and rarely in the ventral horn. Ferritin co-localized with CD68 staining (Fig. 7A–D). Large motor neurons appeared to be devoid of iron as judged by the Perls' DAB stain (Fig. 4D) and ferritin immunoreactivity. Activated microglial cells containing iron were mostly present in the dorsolateral white matter.

## Discussion

We suggest that the T_2_ shortening observed in the motor cortex on MR images of the brain in ALS patients is caused by iron accumulation within microglia. The ferritin-laden microglial cells are primarily found in the middle and deep layers of the motor cortex among the few surviving large pyramidal neurons, amidst reactive astrocytes and cellular debris. This localization coincides with the locations of the cell bodies of corticospinal and callosal neurons [37], [38]. The signal hypointensity was observed on routine clinical T_2_-weighted MRI sequences on a 3T scanner, although it was better localized in the motor cortex on a 7T MRI scanner with T_2_^*^ weighted MRI. The superior spatial resolution and greater sensitivity to paramagnetic metals of 7T MRI was more apparent in the *ex vivo* images which demonstrated signal changes in the deeper layers of the grey matter that corresponded to the localization of increased iron on the tissue histology. Although the MRI signal change was not specific to ALS patients, it occurred disproportionately in ALS patients compared to age-matched controls, in agreement with a previous report [39], and the degree of the signal hypointensity on the 3T FLAIR MRI correlated with the severity of motor impairment as quantified by the UMN-IS and ALSFRS-R.

Iron has a critical role as a cofactor in many regulatory enzymes involved in cellular metabolic pathways, such as the electron transport chain in the mitochondria. It is particularly important in the central nervous system (CNS) due to the high metabolic activity of the brain. Although brain iron content increases with age [12], pathological accumulation of iron is postulated to be pathogenic in several neurodegenerative disorders such as Parkinson disease, neurodegeneration with brain iron accumulation, and Alzheimer disease [40]. Increased iron concentration has also been reported in the ventral spinal cord of ALS patients by laser microprobe mass spectroscopy [15]. Our study showed that iron was also increased in the motor cortex in two ALS patients using histological methods. Even though the motor cortex is known to have the highest concentration of iron in the human cerebral cortex, it is usually undetectable on histologic sections from normal individuals who are less than 65 years old [41], as we also found in our control brain. In our study, iron accumulation in the motor cortex was abundant despite the young age of the two ALS patients who were 42 and 51 years old, and the iron was mainly found in microglia. This localization in the cortical grey differed from previous histologic studies of brain iron in individuals without neurological disorders in which intracellular iron accumulation in the cortex was mostly found in oligodendroglia [42], [43]. Increased iron staining has been reported in the microglia, but mainly in older individuals [12]. We also observed iron accumulation in the motor cortex of a Parkinson disease patient, although the distribution of iron was more diffuse compared to the ALS patients. Although quantitative brain MRI and pathology studies measuring iron content in Parkinson disease patients have shown increased microglial iron in the substantia nigra [44]–[46], iron staining in the motor cortex has received little attention. In the Parkinson control tissue, accumulation of iron in the motor cortex may be related to the rostral progression of neurodegeneration as disease progresses [47]. Additionally, the patient controls were over age 75 and may also have had age-related accumulation of iron [12]. Similar to previous studies of changes in iron content with age, increased iron was not observed in other cortical areas of the brain [41].

It is uncertain what leads to the increased iron deposition in the motor cortex: misregulation of iron metabolism in motor neurons or microglia could cause disease, or microglia could accumulate iron as they scavenge debris that remains after neuronal degeneration. Previous evidence is mixed on the question of whether iron misregulation contributes to ALS pathogenesis. In the CNS, the iron transport protein transferrin is a component of Bunina bodies [48], an intracytoplasmic inclusion in spinal motor neurons that is considered to be a pathological hallmark of ALS. The observation of elevated levels of serum ferritin in ALS patients is thought to indicate a general perturbation of iron metabolism [49], [50]. However, neither serum nor CSF iron levels were found to be consistently elevated [49], [51], suggesting increased ferritin may be a non-specific acute phase reactant in response to inflammatory conditions [52]. While cortical motor neurons are not known to have increased iron levels in ALS, misregulation of iron regulatory proteins leading to accumulation in spinal motor neurons and glia was seen in the G37R SOD1 mutant mice [17]. Neurodegeneration and iron accumulation in oligodendroglia were also seen in mutant mice with targeted deletions of iron regulatory protein-2 [40], [53]. We could not identify iron accumulation within cortical or spinal motor neurons in our patient, which may indicate that if iron misregulation is the primary pathology, its origin is in non-neuronal cells. Of note, there was also no evidence of systemic iron overload in our ALS patients.

Activated microglial cells are a prominent component in both acute CNS injuries and chronic neurodegenerative disorders, including ALS [54]. An important function of the microglia is phagocytosis of cellular debris after neuronal injury, and this has been suggested to provide a more favorable environment to facilitate the repair process [55]. Consistent with their role in removal of degenerated tissue, many microglial cells were seen in the motor cortex and spinal cord where degenerating motor neurons and their axons are found. Iron and microglia are known to accumulate at the sites of neurodegeneration in Alzheimer and Parkinson disease [56]. Microglial iron accumulation has been reported in the striatum of the transgenic mouse model of Huntington disease [57] and in the leukocortical lesions of multiple sclerosis patients [58]. The iron laden microglial cells in our ALS patients were mainly found in the deeper layers of the motor cortex. The association between regions with degenerating neurons and microglial iron accumulation may be related to microglia's role in the maintenance of brain iron homeostasis by scavenging excess iron and storing it in ferritin [59]. Others have postulated that enhanced microglial iron accumulation results in pathological function of these cells in disorders such as multiple sclerosis [60]. We favor the interpretation that the intracellular iron in the microglial cells is derived from phagocytosis of degenerated neurons and glial cells. It is uncertain however whether the initiating event includes a perturbation in the intracellular iron status of these cells prior to their demise.

The correlation between clinical ratings of motor impairment and the grade of hypointensity in the motor cortex suggests a relationship between iron accumulation and progressive neuronal degeneration. In our ALS patient cohort, the motor cortex hypointensity in the 3T FLAIR MRI images was more frequently graded as mild than marked. This may reflect a selection bias for ALS patients earlier in the course of disease who were able to tolerate the scanning procedures. Motor cortex hypointensity in the FLAIR sequence on 3T MR images was present in some healthy controls with normal neurological examinations, although it was mostly graded as mild and rarely graded as marked. Some degree of motor cortex hypointensity is an age related phenomenon and unrelated to neurological disease [8], [9]. Interestingly, iron accumulation in the basal ganglia of rhesus monkeys has been shown to correlate with declining motor abilities independent of age [61]. It could be that the focal excessive iron accumulation in the motor cortex represents a marker of underlying genetic susceptibility for disturbed brain iron regulatory pathways which can lead to cortical motor neuron dysfunction. Compared to other cortical regions, the motor cortex may be particularly vulnerable to changes in iron homeostasis due to its relative high metabolic activity [62] and higher concentration of iron. Additional studies will be necessary to substantiate this hypothesis.

Several different mechanisms have been proposed as the cause of neurodegeneration in ALS including oxidative stress, glutamate induced excitotoxicity, and mitochondrial dysfunction [63]. Iron has been implicated in the pathogenesis of several neurodegenerative disorders due to its ability to generate cytotoxic reactive radicals that produce oxidative stress [64]. However, a pathogenic role in ALS is unclear. Whether intracellular iron accumulation represents an upstream or downstream phenomenon in the disease process remains undetermined. Further understanding of normal iron metabolism in neurons and glial cells is necessary to determine if aberrant iron regulation contributes to pathology in ALS. The hypointensity in FLAIR images, albeit not specific for ALS, may be able to serve as an imaging marker of corticospinal motor neuron degeneration in individual patients over time or predict disease progression, as has been suggested in multiple sclerosis [60]. A limitation of this study was the availability of motor cortex pathology in only two of the ALS patients who were imaged. Longitudinal studies of a larger cohort of ALS patients and controls are needed to determine whether the grading of hypointensity on T_2_-weighted MRI increases as degeneration progresses and whether it represents progressive iron accumulation.
